# Venom Immunotherapy in Clinical Practice: A Retrospective Real-World Cohort

**DOI:** 10.3390/toxins18100447

**Published:** 2026-10-15

**Authors:** İsmet Bulut, Zeynep Yegin Katran, Özge Argın, Mustafa Katran, Fatma Çakmak

**Affiliations:** 1 Department of Allergy and Immunology, University of Health Sciences, Süreyyapaşa Training and Research Hospital, Istanbul 34403, Turkey; drismet.bulut@gmail.com (İ.B.);; 2 Department of Emergency Medicine, Tuzla State Hospital, Istanbul 34403, Turkey; 3 Department of Emergency Medicine, Faculty of Medicine, Istanbul Atlas University, Istanbul 34403, Turkey

**Keywords:** hymenoptera venom allergy, venom immunotherapy, anaphylaxis, omalizumab, premedication, *Vespula*, honey bee, real-world data

## Abstract

Venom immunotherapy (VIT) is the only disease-modifying treatment for Hymenoptera venom allergy, but real-world data describing VIT-related reactions and individualized premedication practices remain limited. We retrospectively evaluated 16 adults undergoing VIT with a target maintenance dose of 100 µg administered every 4 weeks at a tertiary allergy center between January 2024 and December 2025, focusing on clinical characteristics, premedication, treatment-related reactions, and field-sting outcomes during follow-up of up to 24 months. All patients had severe index reactions (Müller grade III-IV); 13 (81.3%) received Vespula VIT and 3 (18.8%) honey bee VIT. Premedication was initiated before VIT in seven patients (43.8%), including omalizumab in five (31.3%). During the 7-week up-dosing phase, five patients (31.3%) developed local reactions, whereas no systemic reactions occurred (0/16; 95% CI: 0–20.6%). During maintenance, one systemic reaction occurred (6.3%), requiring adrenaline and one-night intensive care admission, after which VIT was discontinued. Two patients were re-stung during follow-up and developed only local reactions. In this small, selected real-world cohort, no systemic reactions were observed during the up-dosing phase, whereas one systemic reaction occurred during maintenance. Given the limited sample size, wide confidence intervals, and frequent use of individualized premedication, these findings should be interpreted as descriptive observations and not as evidence establishing the safety of VIT or the effectiveness of premedication.

## 1. Introduction

Hymenoptera venom allergy (HVA) is one of the leading causes of anaphylaxis in adults in Europe and represents a significant proportion of anaphylaxis cases in Türkiye [1,2]. The diagnosis of HVA is established in patients with a compatible clinical history of systemic reaction following a sting together with evidence of sensitization, demonstrated by positive venom-specific IgE (≥0.35 kU/L) and/or positive skin test responses [3].

All patients diagnosed with HVA should be prescribed an adrenaline auto-injector (AAI) due to the risk of recurrent systemic reactions. However, the only disease-modifying treatment for HVA is venom immunotherapy (VIT) [4]. VIT has been shown to significantly reduce the risk of systemic reactions following subsequent stings and is associated with substantial improvement in quality of life [5].

Although double sensitization to both honey bee (*Apis mellifera*) and vespid venom may be detected in diagnostic testing, true double clinical allergy is relatively uncommon in real-life practice. In cases where sensitization patterns are unclear, component-resolved diagnostics may help to identify the clinically relevant venom [6].

When compared with long-term management using only adrenaline auto-injectors, VIT has been shown to be a cost-effective strategy due to its high efficacy in preventing severe systemic reactions [7]. Although adrenaline auto-injectors are essential for the emergency treatment of recurrent systemic reactions, they do not prevent future sting-induced reactions and their effectiveness depends on timely recognition and appropriate administration. Therefore, in patients with an established indication, VIT provides a disease-modifying approach aimed at reducing the risk of systemic reactions to subsequent stings.

Despite the availability of different venom preparations worldwide, real-life data evaluating safety outcomes and premedication strategies with specific commercial products remain limited. In Türkiye, currently available VIT preparations include Roxall^®^, Hymnox^®^, and standardized honey bee and vespid venom extracts. Therefore, this study aimed to describe real-world treatment-related reactions, premedication approaches—including antihistamines, montelukast, and omalizumab—and the clinical course of patients undergoing venom immunotherapy at a tertiary allergy center.

## 2. Results

All 16 consecutive adult patients who underwent VIT during the study period met the eligibility criteria and were included in the analysis. The mean age of the patients was 45.1 ± 15.3 years. Of the 16 patients, 11 (68.8%) were male and 5 (31.2%) were female. Regarding comorbidities, urticaria/angioedema was present in four patients (25%), allergic rhinitis in six (37.5%), atopy in six (37.5%), and asthma in one patient (6.3%). Hypertension was observed in three patients (18.8%). None of the patients were receiving beta-blockers or angiotensin-converting enzyme (ACE) inhibitors, or had a diagnosis of malignancy, mast cell activation syndrome (MCAS), or mastocytosis.

All patients exhibited cutaneous involvement (100%) during the index reaction. Respiratory symptoms were observed in 93.8%, cardiovascular involvement in 75%, and gastrointestinal symptoms in 31.2% of the patients. According to the Müller classification, 4 patients (25%) experienced grade III reactions and 12 patients (75%) experienced grade IV systemic reactions.

Fourteen patients (87.5%) presented to the emergency department following the index reaction, and adrenaline was administered in 13 patients (81.3%). The majority received a single dose of adrenaline, while multiple doses were required in a minority of cases (Table 1).

Venom immunotherapy was initiated predominantly for Vespula venom (81.3%). Baseline serum tryptase levels were within normal limits in most patients. Venom immunotherapy was initiated for honey bee venom in 3 patients (18.8%) and for *Vespula* venom in 13 patients (81.3%). Specific IgE testing revealed sensitization patterns consistent with the selected venom in most patients. Component-resolved diagnostics showed variable positivity for Api m 1, Api m 3, Api m 4, and Api m 10 among honey bee-sensitized patients, while Ves v 1 and Ves v 5 positivity was observed in patients receiving *Vespula* venom immunotherapy. Baseline serum tryptase levels ranged between 1 and 12 ng/mL, remaining within normal limits in the majority of patients. Peripheral blood C-KIT mutation analysis by PCR was negative in all 16 patients. One patient (P7) additionally underwent bone marrow evaluation, which was also negative for a C-KIT mutation. Total IgE levels showed a wide distribution (17–928 IU/mL). Eosinophil counts ranged between 60 and 330 cells/µL, with eosinophil percentages between 0.7% and 3.2%. All patients were prescribed an adrenaline auto-injector (AAI).

During the up-dosing phase, premedication was initiated before VIT in seven patients (43.8%), including omalizumab in five patients (31.3%). Five patients (31.3%) developed local reactions; however, no systemic reactions occurred during up-dosing (0/16; 95% CI: 0–20.6%). Following the development of local reactions, treatment was intensified in three patients: antihistamines and montelukast were added in two patients, and antihistamines alone were added in one patient. The remaining two patients continued their existing premedication regimen without further escalation. No field stings or sting-related reactions occurred during the up-dosing phase.

Sixteen patients entered the maintenance phase. The single systemic reaction during maintenance occurred in a 57-year-old woman receiving Vespula VIT. Her index reaction was Müller grade IV and involved cutaneous, respiratory, cardiovascular, and gastrointestinal manifestations. Her baseline serum tryptase level was 6.6 ng/mL, and she had received omalizumab as premedication before VIT. The maintenance reaction required adrenaline administration and a one-night intensive care unit admission. VIT was subsequently discontinued. Overall, one systemic reaction occurred during maintenance (1/16; 6.3%; 95% CI: 0.2–30.2%). The remaining 15 patients (93.8%) completed at least 12 months of maintenance therapy, and four patients (25.0%) completed 18 months of follow-up. Three patients (18.8%) experienced local reactions during maintenance. Additionally, two patients (12.5%) were re-stung during follow-up and developed only local reactions without systemic involvement. No further systemic reactions were observed during the maintenance period.

## 3. Discussion

In our cohort, *Vespula* venom sensitization was observed in 13 patients, while 3 patients were sensitized to honey bee venom. Although two patients showed sensitization to both venom extracts on conventional specific IgE testing, no patient was considered to have clinically relevant double venom allergy after evaluation of the clinical history and component-resolved diagnostic findings. In real-life clinical practice, genuine double clinical allergy is relatively uncommon [6]. When conventional venom-specific IgE testing is insufficient to identify the culprit venom, component-resolved diagnostics may provide additional diagnostic precision [8,9,10,11]. In two of our patients, positivity to both venom extracts was detected by specific IgE testing; however, component-resolved diagnostics enabled differentiation of the clinically relevant sensitization. Therefore, component-based testing should be considered in selected patients with unclear sensitization profiles.

Careful assessment of comorbidities is essential in patients undergoing VIT, as recommended by current guidelines [4]. All patients in our study were evaluated for conditions that may have influenced VIT safety, including asthma, chronic urticaria, mast cell activation syndrome, and mastocytosis. Notably, all index reactions in our cohort were classified as Müller grade III–IV, indicating severe systemic involvement. In patients presenting with such severe reactions, underlying mast cell disorders should be systematically investigated, as suggested in previous reports [12,13].

One patient developed chronic spontaneous urticaria with concomitant cold urticaria following venom-induced anaphylaxis. Similar cases have been reported in the literature [14,15], suggesting that systemic allergic reactions may act as potential triggers in predisposed individuals.

All patients were treated using a conventional 7-week build-up protocol followed by monthly maintenance injections under strict medical supervision. Post-injection observation for at least two hours was systematically implemented in accordance with safety recommendations [16]. Although rush and ultra-rush protocols have been shown to be safe and effective in selected populations [17,18], we opted for a conventional protocol in this study to ensure optimal safety monitoring.

During the up-dosing phase, only local reactions were observed. One patient experienced a systemic reaction during the maintenance phase, following which VIT was discontinued. Previous studies have reported that adverse events during VIT are generally infrequent and manageable without major interventions [19], which is consistent with our findings.

Premedication strategies were implemented in seven patients prior to VIT initiation, five of whom received omalizumab. Because premedication was individualized according to clinical assessment and no untreated comparator group was available, the present study cannot determine whether omalizumab, antihistamines, or montelukast reduced the risk of VIT-related reactions. Premedication should therefore be regarded as a potential source of confounding by indication, and the observed reaction frequencies should be interpreted descriptively without attributing a protective effect to adjunctive treatment. Omalizumab has been used as an adjunctive strategy in selected patients experiencing systemic reactions during VIT or undergoing accelerated protocols [20], although standardized dosing regimens and treatment durations in this setting have not been established [21]. Accordingly, the outcomes observed in our cohort should not be interpreted as evidence of the effectiveness of omalizumab or other premedication strategies.

Only two patients experienced field stings during follow-up, and both developed local reactions without systemic involvement. Given the very small number of field re-stings, these observations are descriptive and do not permit conclusions regarding the clinical effectiveness of VIT against subsequent stings.

This study has several limitations. First, the small sample size and single-center design limit the precision and generalizability of the findings. Second, the retrospective observational design precludes causal inference. Third, premedication was individualized rather than protocolized, creating substantial potential for confounding by indication; patients receiving omalizumab or other premedication may have differed in baseline clinical risk from untreated patients. Therefore, this study cannot determine whether these interventions reduced VIT-related reactions. In addition, a complete injection-level exposure denominator was not available for the entire cohort; therefore, reaction frequencies are reported at the patient level and should not be interpreted as injection-based incidence rates. As all patients were treated at a tertiary referral allergy center, referral-related selection may further limit generalizability to broader VIT populations. Finally, although follow-up extended up to 24 months in some patients, follow-up duration was heterogeneous, with only four patients completing 18 months of observation. Larger prospective multicenter studies with standardized treatment protocols and injection-level exposure data are needed to better characterize VIT-related reactions and adjunctive premedication strategies. Importantly, the absence of observed systemic reactions during up-dosing should not be equated with evidence of a low underlying reaction risk, particularly given the limited sample size and wide confidence interval. In addition, a complete injection-level exposure denominator was not available for the entire cohort; therefore, reaction frequencies are reported at the patient level and should not be interpreted as injection-based incidence rates. As this cohort comprised consecutive patients treated at a tertiary referral allergy center, referral-related selection may further limit generalizability. Finally, some detailed event-level information was unavailable because of the retrospective design.

Overall, this small descriptive cohort provides a real-world account of conventional VIT in a selected group of patients with predominantly severe Müller grade III–IV index reactions. No systemic reactions were observed during the up-dosing phase, whereas one systemic reaction occurred during maintenance. Given the small sample size, low number of events, wide confidence intervals, and retrospective single-center design, these observations should not be interpreted as establishing the safety of VIT. Rather, they describe the clinical course observed in this selected cohort under specialist supervision and should be considered descriptive and hypothesis-generating.

## 4. Materials and Methods

### 4.1. Study Design and Population

This single-center retrospective cohort study was conducted at a tertiary referral allergy clinic between January 2024 and December 2025. All consecutive adult patients who underwent VIT at our center between January 2024 and December 2025 were assessed for eligibility. All 16 patients met the eligibility criteria and were included in the final analysis; therefore, no patients were excluded after screening. Eligibility required a documented history of a systemic reaction following a Hymenoptera sting together with evidence of sensitization demonstrated by venom-specific IgE levels ≥ 0.35 kU/L. Sensitization to *Apis mellifera* and/or *Vespula* species was considered eligible. Patients with isolated large local reactions without systemic involvement were not considered candidates for VIT.

Patients with isolated large local reactions without systemic involvement were not routinely considered candidates for VIT.

### 4.2. Venom Immunotherapy Protocol

Venom immunotherapy was administered according to international guidelines for Hymenoptera venom allergy. The up-dosing phase consisted of weekly incremental injections over a 7-week period until a target maintenance dose of 100 µg venom was achieved. Following the build-up phase, maintenance injections were administered at 4-week intervals. Patients were followed for up to 24 months during the maintenance phase. All injections were administered in a hospital setting under specialist supervision. After each injection, patients were observed in the hospital for at least 2 h to monitor for potential local or systemic adverse reactions. Any reactions were documented and managed according to standard clinical practice. The detailed dosing schedule is presented in Table 2.

A conventional 7-week build-up protocol was applied using Hymnox^®^ (ROXALL Medicina, Zamudio, Spain), a highly purified, lyophilized venom extract. Extract concentrations of 25 µg/mL and 100 µg/mL were used according to the dose-escalation schedule shown in Table 2. The target maintenance dose was 100 µg, administered every 4 weeks.

### 4.3. Data Collection

Clinical and demographic data were retrospectively extracted from electronic medical records and standardized venom immunotherapy follow-up forms. Recorded variables included age, sex, and comorbid conditions such as asthma, allergic rhinitis, chronic urticaria, atopy, and hypertension. Concomitant beta-blocker use was documented due to its potential impact on systemic reaction severity.

Information regarding the culprit venom (*Apis mellifera* or *Vespula* species), the commercial venom extract used, and detailed characteristics of the immunotherapy protocol were recorded. Component-resolved diagnostics (CRD) were performed selectively in patients in whom conventional venom-specific IgE findings did not clearly distinguish the clinically relevant venom, particularly in those showing sensitization to both honey bee and *Vespula* venom. CRD findings were interpreted together with the clinical sting history and conventional sensitization results to support selection of the venom used for VIT.

As part of the evaluation for underlying mast cell disorders, peripheral blood C-KIT mutation analysis by polymerase chain reaction (PCR) was performed in all 16 patients. One patient (P7) additionally underwent bone marrow evaluation, including C-KIT mutation analysis.

Premedication administered prior to the up-dosing phase or during maintenance was systematically documented. These included antihistamines, montelukast, and omalizumab. The specific agents used and their respective doses were recorded for each patient.

Local and systemic reactions occurring during both the build-up and maintenance phases were retrospectively recorded from medical records and standardized VIT follow-up forms. All patients were observed in the hospital for at least 2 h after each VIT injection. At each subsequent visit, patients were systematically asked whether they had experienced any symptoms or adverse reactions after leaving the hospital following the previous injection, allowing reactions occurring after the observation period to be captured. During the maintenance phase, injection-related adverse reactions and interval field-sting exposures were also systematically documented. During the maintenance phase, patients were followed longitudinally for up to 24 months. Monthly follow-up data included injection-related adverse reactions as well as field sting exposure between visits. In cases of field sting, the presence and severity of any resulting reaction were documented.

### 4.4. Statistical Analysis

Statistical analyses were performed using IBM SPSS Statistics for Windows, version 25.0 (IBM Corp., Armonk, NY, USA). Given the small sample size and descriptive nature of the study, no formal between-group hypothesis testing was performed. Continuous variables were summarized using mean ± standard deviation or median (interquartile range), as appropriate, and categorical variables were presented as frequencies and percentages. Exact 95% confidence intervals were calculated for the observed patient-level frequencies of systemic reactions during the up-dosing and maintenance phases. No imputation was performed for missing data; descriptive analyses were based on available information in the retrospective records.

This retrospective study was approved by the University of Health Sciences, Süreyyapaşa Chest Diseases and Thoracic Surgery Training and Research Hospital Scientific Research Ethics Committee, on 24 February 2026 (protocol code: 2024-152). The approval covered the retrospective analysis of clinical data collected between January 2024 and December 2025.

## Figures and Tables

**Table 1:** Patient-level clinical characteristics, premedication, and outcomes during venom immunotherapy.

Pt	Age/Sex	Culprit Venom	Müller	Index Reaction	Tryptase	Sensitization/CRD	Premedication Before VIT	Up-Dosing Reaction	Maintenance Duration	Maintenance Reaction	Field Sting	Final Outcome
P1	38/M	Honey bee	IV	C + R + CV + GI	8.6	Honey bee sIgE positive	None	None	>12 mo	None	None recorded	Ongoing
P2	61/M	Honey bee	IV	C + R + CV + GI	5.6	Honey bee sIgE positive Api M1: 0.73 Api M10: 1.48	None	None	>12 mo	Local reaction	None recorded	Ongoing
P3	57/F	Vespula	IV	C + R + CV	5.0	Vespula sIgE positive	None	Local	>12 mo	None	None recorded	Ongoing
P4	25/M	Vespula	III	C + R	6.6	Vespula sIgE positive	None	Local	>12 mo	None	None recorded	Ongoing
P5	45/M	Vespula	III	C + R	2.0	Vespula sIgE positive	None	None	>12 mo	None	Field sting recorded	Ongoing
P6	57/F	Vespula	IV	C + R + CV + GI	6.6	Vespula sIgE positive Ves V1:0.5 Ves V5:0.5	Omalizumab	None	Discontinued	Systemic reaction	None recorded	Discontinued after reaction
P7	61/M	Vespula	IV	C + R	12.0	Vespula sIgE positive Ves V1:2.17 Ves V5:1.91	Omalizumab 1 month apart before up dosing	None	>12 mo	None	None recorded	Ongoing
P8	40/F	Vespula	IV	C + R + CV	5.0	Vespula sIgE positive VesV1: 4.9 Ves V5: 37	Omalizumab 300 mg every 28 days	Local	>12 mo	Local	--	Ongoing
P9	37/M	Vespula	IV	C + R + CV	5.4	Vespula sIgE positive Ves V1:0.1 Ves v5:0.17	Omalizumab 300 mg every 28 days	Local	>12 mo	None	None recorded	Ongoing
P10	68/M	Vespula	IV	C + R + CV	5.0	Vespula sIgE positive Ves V1:100	Antihistamine	None	>12 mo	None	None recorded	Ongoing
P11	19/M	Vespula	IV	C + CV	1.6	Vespula sIgE positive Ves V1:2.6 Ves V5: 2.1	Antihistamine	None	>12 mo	None	None recorded	Ongoing
P12	59/F	Vespula	III	C + R + GI	5.0	Vespula sIgE positive Ves V5: 2.1	None	None	>12 mo	Local	None recorded	Ongoing
P13	23/M	Vespula	IV	C + R + CV	5.5	Vespula sIgE positive	None	None	>12 mo	None	--	Ongoing
P14	46/F	Vespula	IV	C + R + CV	3.0	Vespula sIgE positive	Omalizumab 300 mg every 28 days	Local	>12 mo	Local	--	Ongoing
P15	55/M	Vespula	IV	C + R + CV + GI	3.1	Vespula sIgE positive	None	None	>12 mo	None	None recorded	Ongoing
P16	31/M	Vespula	IV	C + R + CV	1.0	Vespula sIgE positive	None	None	>12 mo	None	None recorded	Ongoing

Abbreviations: Pt, patient; M, male; F, female; C, cutaneous; R, respiratory; CV, cardiovascular; GI, gastrointestinal; CRD, component-resolved diagnostics; sIgE, specific immunoglobulin E; VIT, venom immunotherapy; mo, months; mg, milligrams.

**Table 2:** Conventional 7-week build-up and maintenance protocol for venom immunotherapy (Hymnox^®^).

Week	Concentration (µg/mL)	Volume (mL)	Administered Dose (µg)
Day 1	25 µg/mL	0.1	2.5
Day 7	25 µg/mL	0.2	5
Day 14	25 µg/mL	0.4	10
Day 21	25 µg/mL	0.8	20
Day 28	100 µg/mL	0.4	40
Day 35	100 µg/mL	0.6	60
Day 42	100 µg/mL	0.8	80
Day 49	100 µg/mL	1.0	100
Maintenance	100 µg/mL	1.0	100

## Data Availability

The data presented in this study are available from the corresponding author upon reasonable request. The data are not publicly available due to patient privacy and ethical restrictions.
